# Self-Assembled MXene/MWCNTs Pressure Sensors Combined with Novel Hollow Microstructures for High Sensitivity

**DOI:** 10.3390/mi17010003

**Published:** 2025-12-19

**Authors:** Zhicheng Wang, Hongchen Yu, Xingyu Ma, Yijian Liu, Fei Wang, Da Chen

**Affiliations:** Laboratory for Intelligent Flexible Electronics, College of Electronic and Information Engineering, Shandong University of Science and Technology, Qingdao 266590, China; wangzhicheng320@163.com (Z.W.); yuhc0207@163.com (H.Y.); mxy18253103525@163.com (X.M.); liuyijian@sdust.edu.cn (Y.L.)

**Keywords:** hollow microstructure, self-assembled MXene/MWCNTs, movement detection, machine learning

## Abstract

Flexible pressure sensors have garnered significant attention over the past few decades owing to their indispensable role in electronic skin and health monitoring, and there is an urgent demand for high sensitivity to meet the requirements of large-scale applications. In this work, we demonstrate a resistive pressure sensor with self-assembled MXene/MWCNTs complex conductive networks, whose hollow substrate is achieved via designed molds and thermally expandable microspheres. Herein, the pressure sensor exhibits the desired performances, including a high sensitivity of 2.63 kPa^−1^, an ultra-low detection limit of ~0.25% relative resistance change, and rapid response times of 340 ms. The high performance enables promising prospects for detecting diverse human body movements. More importantly, it has been applied in numerical classification based on machine learning with the Hidden Markov Model, achieving an impressive accuracy of ~99.2%. Our research offers novel insights for enhancing the performance of pressure sensors, which hold great potential for practical applications.

## 1. Introduction

Flexible pressure sensors with high sensitivity, a wide sensing range, and excellent durability hold substantial potential in electronic skin, health monitoring, and human–computer interaction, which have continuously driven device innovations over the past few decades [1,2,3]. Nevertheless, existing sensors still face challenges in balancing sensing range, sensitivity, and fabrication processes [4,5,6,7]. However, these limitations could be overcome via novel material combinations, substrate modifications, and fabrication designs.

Numerous efforts have been devoted to novel material combinations, in which advanced materials with perfect conductivity are utilized in flexible sensors, including metal nanoparticles [8,9,10], carbon nanotubes [11], reduced graphene oxide [12], and MXene [13,14,15]. Thereinto, MXene materials with a layered 2D structure are regarded as promising candidates because of their high conductivity, better biocompatibility, and outstanding mechanical flexibility, which could achieve high sensitivity for MXene-based sensors, but narrow response range remains a significant challenge [16,17,18]. Owing to their superior mechanical strength and stable properties, one-dimensional (1D) multi-walled carbon nanotubes (MWCNTs) have attracted considerable attention, which are critical for extending sensing range and stability for flexible sensors [19,20,21]; however, their sensitivity seems to be slightly inadequate for practical applications. Fortunately, novel material combinations used as conducting layers could effectively overcome their inherent restrictions. Thus, MXene/MWCNT composite materials are frequently used in sensors to achieve high sensitivity and a wide sensing range [22,23,24,25]. Self-assembled MXene/MWCNT composite networks constitute an effective strategy for enhancing sensor performance, which has garnered considerable attention due to their simple fabrication process; however, further investigation is required to facilitate their extensive applications. Another key point is that the microstructural processing of the substrate has been employed in previous works to achieve designed morphologies, such as micro-patterned substrates, porous substrates, and multi-microstructured substrates [26,27,28,29], which has proven effective in enhancing pressure sensor performance. In recent advances, the integration of geometrically microstructured substrates into flexible pressure sensors, including pyramid, hemispherical, and cylindrical structures, has become a common and effective strategy [30,31]. The sharp structure could distinctly amplify contact signals under tiny pressures, while the spherical surface is conducive to realizing a wide sensing range. Moreover, solid microstructures only provide limited sensitivity, whereas hollow structures lead to a promising way for high sensitivity over a broad pressure range [32]. Accordingly, the hollow substrates with nested semispherical/pyramid structure are expected to play a crucial role in ensuring impressive sensing performance. Several reports have focused on MXene/MWCNTs or hollow substrates for high-performance pressure sensors [33,34]. Most research aims at fabricating monolayer MXene or modifying MWCNTs materials for functional modifications, but the complex fabrication process restricts their technical application. As for hollow substrates, to achieve a desirable balance between sensing range and sensitivity, further investigations are still required for geometrical designs and hollow structure achievements. Furthermore, systematic studies on self-assembled MXene/MWCNTs pressure sensors with hollow microstructure substrate remain largely unexplored. In addition, there are few detailed analyses revealing the synergetic mechanism. Thus, it is urgently required to reveal the mechanism systematically, which is pivotal for potential applications of flexible pressure sensors.

In this work, we systematically studied resistive pressure sensors with self-assembled MXene/MWCNTs conductive networks and hollow microstructured flexible substrates, in which MXene particles are interconnected by MWCNTs to form complex conductive networks, while hollow microstructures are constructed via designed molds and thermally expandable microspheres, which further complicate substrates for impressive performance. It is found that our sensors exhibit high sensitivity (2.63 kPa^−1^), a rapid response time of 340 ms, and a low detection limit of ~0.25% relative resistance change, which enable reliable human body detection and numerical classification with an impressive accuracy of 99.2%. Our results provide a novel method for boosting the performance of pressure sensors, thereby facilitating their compliance with the rigorous performance requirements of next-generation wearable electronic devices.

## 2. Experimental

### 2.1. Materials and Preparations

The MXene (Ti_3_C_2_T_x_) was purchased from Guangdong Foshan Xinxi Technology Company Limited, Foshan, China. The MWCNTs, whose diameters are 3–15 µm and average lengths are 15–30 μm, were provided by Hongdakang Evolution Technology Company Limited, Shenzhen, China. Sodium dodecyl sulfate (SDS) was purchased from Kena Carbon New Materials Company Limited, Xiamen, China. Polydimethylsiloxane (PDMS) was purchased from Dow Corning Company Limited, Midland, MI, USA. The thermally expandable microspheres (TEMs) were provided by Jiasheng Advanced Materials Technology Company Limited, Shenzhen, China. Poly(diallyldimethylammonium chloride) (PDDA) and poly(sodium 4-styrenesulfonate) (PSS with ~70,000 average molecular weight) were provided by Wuxi Tianxin Chemical Company Limited (Wuxi, China) and Shanghai Yuanye Biotechnology Company Limited (Shanghai, China), respectively.

**Figure 1:**
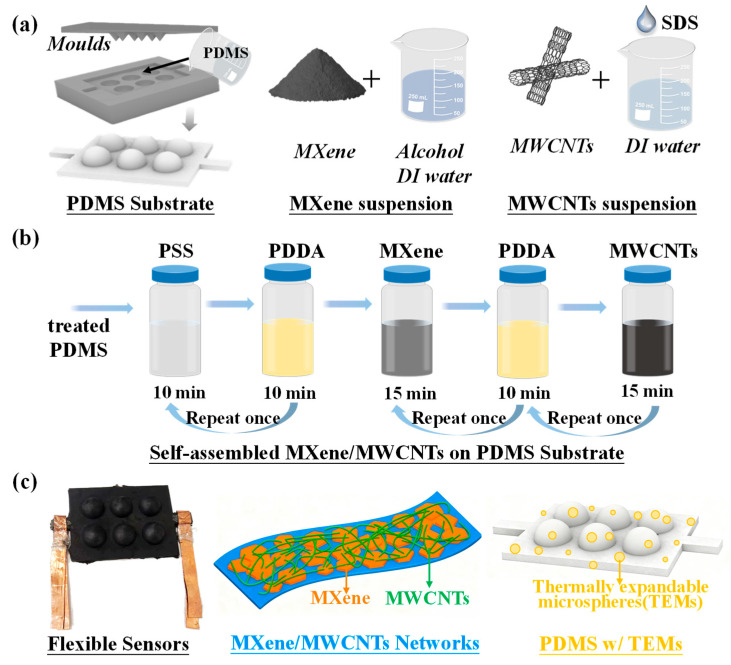
Fabrication of self-assembled MXene/MWCNT/PDMS pressure sensors. (**a**) PDMS substrate fabrication by molds, preparation of MXene and MWCNTs suspensions; (**b**) illustration of the self-assembly method for conductive layers; (**c**) image of flexible sensor with the schematic of MXene/MWCNTs conductive networks and hollow PDMS substrate.

Based on these materials, substrates and suspensions were prepared for the fabrication of self-assembled pressure sensors. As for the substrates in Figure 1a, 10 g PDMS and 1 g curing agent (Sylgard 184 Silicone Elastomer Kit) were mixed thoroughly in beakers, followed by the incorporation of TEMs at a weight percentage (wt%) of 0.0–1.5%. The homogenized mixture was poured into molds and cured at 65 °C for 3 h to solidify the PDMS substrates. Subsequently, the PDMS substrates were baked at 100 °C for 3 min to induce the expansion of TEMs, thereby forming hollow microstructures. Afterwards, oxygen plasma treatment was applied on PDMS substrates for 30 s to enhance their ionic absorptivity. The preparation process of suspensions is illustrated in Figure 1a. To obtain Ti_3_C_2_T_x_ suspensions, 2 g Ti_3_C_2_T_x_ powder and 4 mL absolute ethanol were taken into a mortar for 15 min grinding; then, the resulting abrasive was mixed with 46 mL absolute ethanol and 50 mL deionized water. Concurrently, 2 g MWCNTs powder and 1.5 g SDS dispersant were added into 100 mL of deionized water. Finally, the sonication was utilized continuously to ensure the Ti_3_C_2_T_x_ and MWCNTs suspensions uniformly. 1.5 g PDDA was taken into deionized water for a 1.5 wt% PDDA solution, and 0.5 wt% PSS solution was also prepared for subsequent self-assembly process.

### 2.2. Fabrication of Self-Assembled MXene/MWCNTs/PDMS Sensors

The self-assembly fabrication process of MXene/MWCNTs/PDMS pressure sensors is illustrated in Figure 1b. Firstly, the plasma-treated PDMS substrates were immersed in a PSS solution for 10 min and then transferred to beakers for drying; the same treatment was carried out in PDDA solution. Subsequently, repeat the above process once again to obtain PSS/PDDA films. Hence, the surface charge possessed by PSS and PDDA could be competent for capturing more ions for self-assembly, in which the PDDA carries positive charges for attracting negatively charged materials such as MXene and MWCNTs, and the PSS could adsorb enough positive charge. Subsequently, the self-assembled process was performed as follows: the substrates with PSS/PDDA films were immersed in MXene suspensions for 15 min and then dried to form self-assembled MXene films. Next, 10 min immersion in the PDDA solution followed by drying was utilized for preferable adsorption of the next self-assembled MWCNT films; afterwards, the same treatment in MWCNTs suspensions and PDDA solution, as above, was employed. To date, one layer of MXene/MWCNTs was complete, and the repeated process was necessary to form self-assembled MXene/MWCNTs conducting layers. For sensor electrode integration, copper foils were attached using silver paste to ensure reliable electrical contact, and the polyurethane tape was applied to fasten electrodes. To protect the sensing layers and further reinforce the electrode connections, additional PDMS with curing agent was employed to form package layers. Thus, a self-assembled MXene/MWCNTs/PDMS pressure sensor was fabricated, as depicted in Figure 1c, with a schematic diagram attached for further comprehension. Wherein, the MXene particles are interconnected by MWCNTs to form robust conductive networks, while hollow microstructures were constructed via designed molds and TEMs further complicating the substrates for impressive performance.

### 2.3. Characterization of Materials and Devices

Scanning electron microscopy (SEM, TESCAN MIRA LMS, Brno, Czech Republic) is used to characterize the MXene/MWCNTs conductive networks and PDDA/PSS-coated PDMS. X-ray photoelectron spectroscopy (XPS, ESCA-3400, Kyoto, Japan) is performed to prove the successful adhesion of PDDA/PSS on PDMS. Raman spectra of self-assembled MXene/MWCNTs, MWCNTs and MXene materials are obtained by Raman spectrometer (Horiba LabRAM HR Evolution, Horiba, Japan). High-precision digital multimeter (Rigol DM3068 6.5 Digits, Suzhou, China) and data acquisition instrument (Keysight DAQ970A, Santa Rosa, California, USA) constitute the test platform for the performance measurements of MXene/MWCNTs/PDMS pressure sensors. The relative resistance change and sensitivity of the resistive pressure sensor are calculated as follows, respectively:

(1) $$\frac{\Delta R}{R_{0}}=\frac{R-R_{0}}{R_{0}}$$

(2) $$S=\frac{\delta \left(\Delta R/R_{0}\right)}{\delta P}$$

where *R* is the resistance of sensors under the pressure condition; *R_0_* is their initial resistance; and *P* is the change in the external pressure.

## 3. Results and Discussion

**Figure 2:**
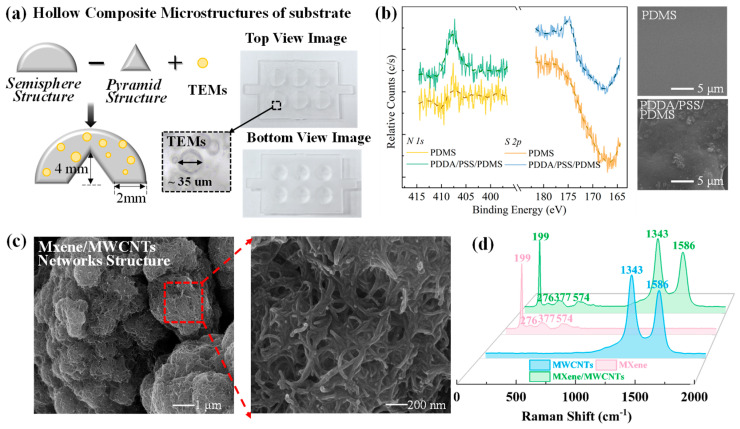
(**a**) Schematic diagram and optical images of the hollow PDMS substrate incorporated with thermally expandable microspheres (TEMs), with an enlarged inset showing the detailed morphology of hollow microspheres; (**b**) XPS and SEM of native PDMS and PDDA/PSS-coated native PDMS; (**c**) SEM of MXene/MWCNTs composite conductive networks, with a magnified inset illustrating the local microstructural features; (**d**) Raman spectra of MWCNTs, MXene, and the self-assembled MXene/MWCNTs composite layers.

A schematic of hollow substrate is displayed in Figure 2a to illustrate the mechanism clearly. Considering that the sharp structure could significantly amplify contact signals under tiny pressures, while the spherical surfaces facilitate a wide sensing range, a hollow substrate with nested semisphere/pyramid structures was adopted in this research. In addition, the thermally expandable microspheres (TEMs) were also utilized for further complicating the geometric hollow substrate. Top and bottom view SEM images of the substrate (Figure 2a) reveal its macroscopic physical structure, while the magnified inset image illustrates the morphology of TEMs-derived hollow microspheres (~35 μm in diameter). The complex hollow microstructure substrates contribute to providing an interior cavity for more space under compression, which could be favorable for sensors’ performance. The self-assembled MXene/MWCNTs conductive layers play a pivotal role in determining the sensor performance, wherein the PDDA/PSS serves as a critical intermediate for self-assembly process. Hence, the oxygen plasma treatment was performed to modify the poor adhesion of PDMS, which is further proved by the XPS and SEM as shown in Figure 2b. The XPS spectrum exhibits characteristic peaks of N 1s and S 2p at ~407 eV and ~174 eV, respectively, which are attributed to the achievement of PDDA/PSS-coated PDMS [35]. In addition, compared with the SEM images of native PDMS, the obvious film could be observed on the PDDA/PSS/PDMS surface, further verifying the successful adhesion of PDDA/PSS on plasma-treated PDMS. The top-view SEM images of MXene/MWCNTs conductive layers are shown in Figure 2c, where well-interconnected conductive networks are distinctly visualized. It is found that MXene particles are wrapped and bridged by dense MWCNTs, and the MWCNTs serve as conductive bridges to connect MXene particles, thereby achieving a robust composite conductive network via a self-assembly process. In Figure 2d, the Raman spectroscopy is shown for further illustration, in which the MWCNTs possess 1336 cm^−1^ and 1573 cm^−1^ primary peaks and MXene provides four significant peaks at 199, 276, 377, and 574 cm^−1^. Moreover, as for the MXene/MWCNTs film, there are all the feature peaks same as those of respective MXene and MWCNTs, which demonstrates that homogeneous MXene/MWCNTs conductive networks are successfully constructed via self-assembly. These results indicate that the hollow microstructured PDMS substrates and self-assembled MXene/MWCNTs uniform conductive networks could be achieved for the realization of high-performance sensors.

**Figure 3:**
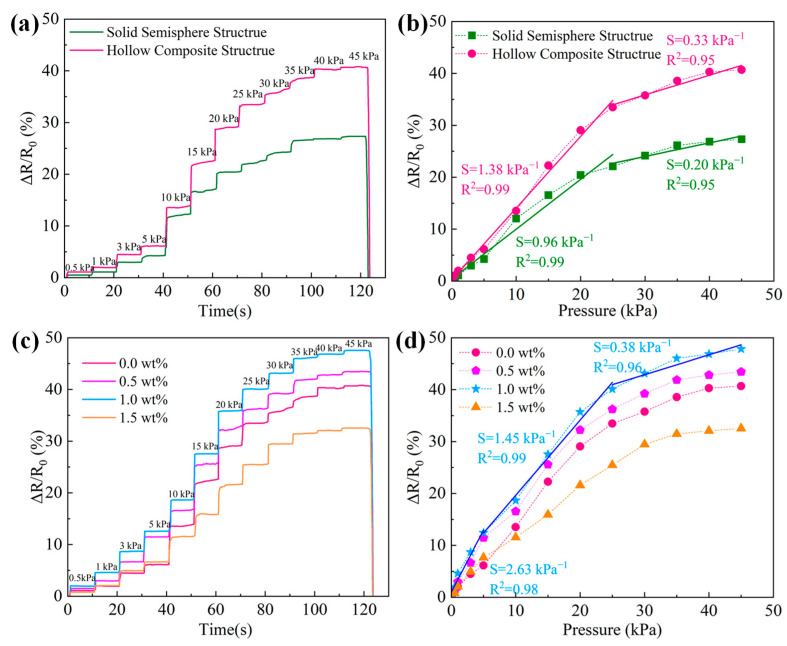
(**a**) Real-time relative resistance change in sensors with solid and hollow substrates under incremental pressure; (**b**) corresponding sensitivity curves (dashed lines) and linear fitting curves (solid lines); (**c**) relative resistance changes in hollow microstructure sensors with varying TEMs (0.0~1.5 wt%) under incremental pressure; (**d**) corresponding sensitivity curves (dashed lines) and linear fitting curves (solid lines).

To comprehensively investigate the effects of hollow substrate on sensor performance, the real-time relative resistance change and sensitivity curves are presented in Figure 3, in which hollow substrates are constructed via designed molds and thermally expandable microspheres. Obviously, the preferable performance could be obtained with a wide pressure range of 0–45 kPa, where the synergetic effect of material combinations and substrate modifications makes significant contributions. As for self-assembled conductive layers, the MXene particles are surrounded and bridged by dense MWCNTs forming a robust interconnected network, which is beneficial to the wide pressure range. For comparative analysis, sensors with identical self-assembled MXene/MWCNT conductive layers but different substrate structures (solid and hollow structure, both fabricated via designed moulds) were characterized and their performance is depicted in Figure 3a,b. Compared with solid semispherical substrates, the hollow substrates with nested semispherical/pyramid structure provides a greater compressive deformation quantity under tiny pressure, yielding a larger relative resistance change, as shown in Figure 3a. As illustrated in Figure 3b, a consistent trend is observed in corresponding sensitivity curves, where the pressure sensor demonstrates two distinct sensitivity characteristics. In the low-pressure range (0–25 kPa), the sensitivity is significantly enhanced from 0.96 kPa^−1^ (*R^2^* = 0.99) for solid structure sensor to 1.38 kPa^−1^ (*R*^2^ = 0.98) for hollow structure sensor. The complex hollow structure substrates contribute to providing an interior cavity for more space under compression, which could be favorable for sensor performance. In addition, as the applied pressure increased, the sensitivity decreased rapidly within the high-pressure range (25–45 kPa). The saturated deformations of solid structure are realized under high pressure, leading to a sensitivity of 0.2 kPa^−1^ (*R*^2^ = 0.95), and the hollow sensors retain a relatively higher sensitivity of 0.33 kPa^−1^ (*R*^2^ = 0.95), which might be due to the exhaustion of compressive deformation under high pressure. Briefly, the designed-molded hollow structure introduces internal cavities that contribute to the high sensitivity of pressure sensors, which provides an effective strategy for boosting the performance of flexible pressure sensors. Additionally, the effect of TEMs on sensor performance has been systematically investigated, where the relative resistance changes and sensitivities of hollow structure sensors with 0.0, 0.5, 1.0, and 1.5 wt% TEMs are elaborated in Figure 3c. After a 100 °C bake, as an additive, the TEMs expand to hollow microspheres, forming complex microstructures within PDMS substrates. Considering the tiny amount of doping, the mechanical and dielectric properties of PDMS are regarded as unchangeable. Based on measured results, it is evident that the TEMs could impact sensor performance effectively, in which the relative resistance changes are enhanced gradually with the increment of the additive. The resistance variations arising from diverse ratios could be attributed to the complex microstructure induced by the synergistic effect of TEMs and the hollow geometric structure. In addition, the sensitivity curves in Figure 3c,d exhibit three characteristic pressure ranges (0–5, 5–25, and 25–45 kPa). Notably, the sensor with 1.0 wt% TEMs achieves an impressive sensitivity of 2.63 kPa^−1^ (*R*^2^ = 0.98) in the lower-pressure range of 0–5 kPa. Compared with the contributions of hollow geometric structure, the TEMs-derived microstructure is regarded as the primary reason for high sensitivity. In the range of 5–25 kPa, a sensitivity of 1.45 kPa^−1^ (*R*^2^ = 0.98) is obtained, wherein the hollow geometric structure is dominant for device performance. Under 25–45 kPa pressure, the exhausted compressive deformation results in a reduced sensitivity of 0.38 kPa^−1^ (*R*^2^ = 0.98); however, it is still higher than that of sensors with single hollow geometric structure, which demonstrates the effectiveness of TEMs in performance improvement intuitively. Meanwhile, the sensor with 1.5 wt% TEMs exhibits degraded performance, which is presumably due to excessive TEMs doping causing the poor properties of hollow substrates. Consequently, an optimal dosage of TEMs could effectively optimize the sensor performance; whereas excessive additives are detrimental to sensing behavior. Synthetically, the pressure sensors with promising sensitivity and wide sensing range could be attributed to the synergetic mechanism of self-assembled MXene/MWCNTs and novel hollow microstructures via designed moulds and TEMs. To comprehensively evaluate the sensor performance, a comparison table of relevant flexible pressure sensors is summarized in Table 1 [36,37,38,39,40,41,42]. It is evident that our sensors possess a clear advantage in sensitivity, which illustrates that the combination of self-assembled MXene/MWCNTs networks and hollow microstructures provides an efficient strategy for the pressure sensors.

**Figure 4:**
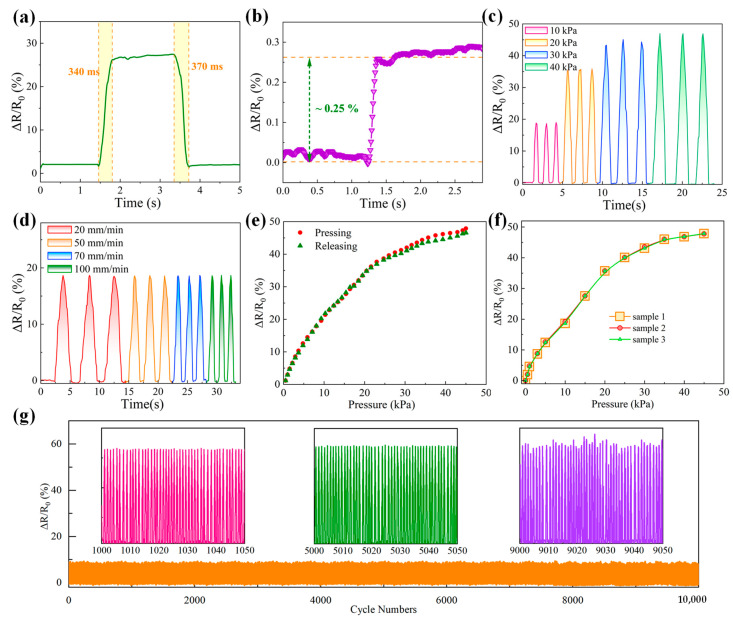
(**a**) Response and recovery times under a compressive deformation of 1 mm; (**b**) relative resistance change under a minimal pressure of 10 mN; (**c**) cyclic variation in the relative resistance as a function of different pressure levels (10~40 kPa); (**d**) relative resistance change under diverse cyclic measurements at varying speed with ~10 kPa pressure; (**e**) real-time relative resistance response curves during pressing and releasing cycles; (**f**) relative resistance changes of 3 samples to verifying the consistency of sensor performance; (**g**) long-term durability test results with 10,000 cyclic pressing and releasing processes under an applied pressure of ~3 kPa.

To further investigate the practical applicability of self-assembled MXene/MWCNT hollow pressure sensors, systematic performance characterizations were conducted on the sensors based on a 1.0 wt% ratio of TEMs, as presented in Figure 4. In Figure 4a, the pressure sensors exhibit response and recovery time of 340 ms and 370 ms, respectively, under the deformation quantity of 1 mm. The response–recovery times are relatively long for pressure sensors, which are presumably attributed to the full package on flexible sensors. Fortunately, these sensors are still suitable for practical applications, including real-time movement detections and healthcare monitoring. The ability of pressure sensors to detect tiny pressure signals is explored as shown in Figure 4b, where 10 mN pressure is utilized on sensors; ~0.25% ΔR/R_0_ is achieved, proving the sensor’s feasibility for weak signal detection. The real-time relative resistance changes with diverse pressure (10 to 40 kPa) for 3 cyclic pressing and releasing are presented in Figure 4c. Consistent and regular fluctuations are obtained under the same pressure, while the response signals distinguish with different pressure due to diverse deformation, indicating that the sensor could recognize various pressure levels and provide a preferable and stable sensing response without signal attenuation. To reveal the effects of testing speed (controlled by experimental test platform tensile machine) on sensing performance, cyclic measurements are performed at varying speeds from 20 to 100 mm/min under ~10 kPa pressure, and the corresponding results are displayed in Figure 4d. It is clearly seen that the sensors’ response–recovery time varies with different testing speeds, where the response speed is enhanced with the elevation of testing speed. Moreover, electrical signals’ value remains essentially unchanged at diverse speeds, whose consistency further illustrates the favorable stability of the sensors. The real-time relative resistance change curves under pressing–releasing are shown in Figure 4e; the highly consistent response profiles indicate a high recoverability with tiny hysteresis errors. As illustrated in Figure 4f, accordant ΔR/R_0_ responses of three samples were obtained, verifying the consistency of sensor performance, where samples 1 and 2 are within the same batch and sample 3 belongs to another batch. The repeatability of pressure sensor is further validated via long-term durability measurements under an applied pressure of ~3 kPa in Figure 4g. After 10,000 cyclic pressing and releasing cycles, the response signal amplitude remained practically constant. Meanwhile, the magnified insets display the ΔR/R_0_ variations in detail, where a slight increase in resistance fluctuation is observed at the later stage of cycling, but it is still a negligible signal change in consideration of mechanical losses. This impressive long-term durability and stability illustrate the synergistic advantage of self-assembled MXene/MWCNTs and hollow microstructured substrates indirectly. Considering high linearity, minimal hysteresis error, and preferable repeatability, high sensor accuracy could be achieved in our pressure sensors. Therefore, the self-assembled MXene/MWCNTs pressure sensors combined with novel hollow microstructures possess high sensitivity, a wide pressure detection range, rapid response time, and long-term stability, rendering them well-suited for the practical requirements of flexible sensing applications.

**Figure 5:**
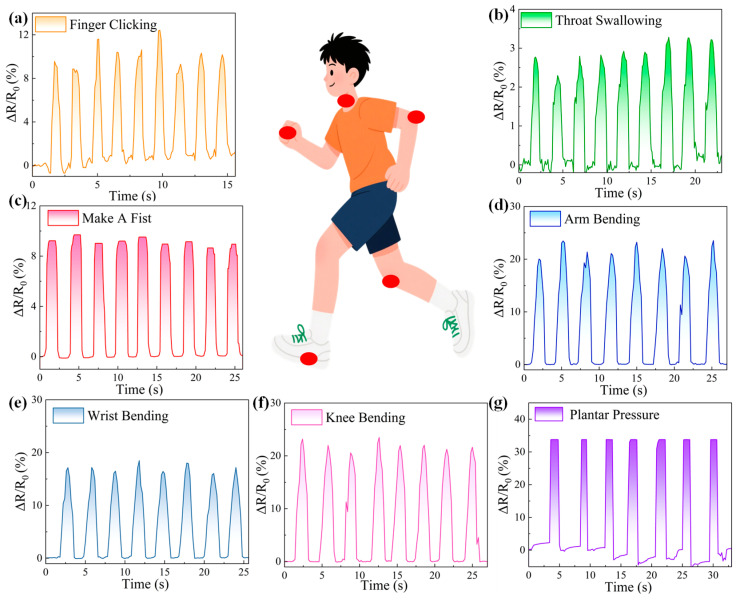
Real-time relative resistance change (ΔR/R_0_) responses of sensors during various human motions: (**a**) finger clicking; (**b**) throat swallowing; (**c**) making a fist; (**d)** arm bending; (**e**) wrist bending; (**f**) knee bending; (**g**) plantar pressure. The sensors were attached to the corresponding body positions marked by red circles in the schematic illustrations.

Considering the sensor’s promising performance, the hollow microstructured MXene/MWCNT pressure sensors are well-suited for human motion detection. As depicted in Figure 5, the relative resistance change (ΔR/R_0_) responses were measured with motion of finger clicking, throat swallowing, making a fist, arm bending, wrist bending, knee bending, and plantar pressure. These results verify the multifunctional sensing capability of sensors and produce a reliable database for health monitoring. Figure 5a demonstrates the sensor’s response characteristics of finger clicking, where the obvious output signals confirm the detection capability for dynamic local pressure variations. Notably, benefiting from the ultra-low detection limit of ~0.25% relative resistance change, subtle movements of human body could be detected effectively. For instance, as shown in Figure 5b, a sensor attached to the throat successfully captured small ΔR/R_0_ changes induced by swallowing motions. Subsequently, a sensor was affixed on the opisthenar to monitor the movement of making a fist, with obvious response signals displayed in Figure 5c. These results demonstrate that the pressure sensor could capture the contraction and relaxation states of the hand muscles effectively. In addition, the maximum value of clenching fist signals exhibits a flat tendency instead of a sharp value, which could be attributed to the pressure applied on the whole sensor plane for several seconds during muscle contraction. The electrical signals generated by arm bending are clearly depicted in Figure 5d, in which our sensor is adhered to the arm elbow. Significant ΔR/R_0_ values were observed due to the large movement amplitude of the elbow, which reflects the dynamic elbow activity and offers broad application potential in health monitoring. Analogously, the real-time monitoring signals of wrist bending and knee bending are shown in Figure 5e,f, where the sensor accurately captures these movements and delivers feedback effectively. For wrist bending, the limited movement range leads to moderate relative resistance changes, which still prove the effectiveness of our pressure sensors in movement detection. During knee flexion, consistent and regular fluctuations are recorded, offering a reliable database for evaluating knee joints. Figure 5g illustrates the sensors’ performance in plantar pressure detection, where the stable response signals indicate the capability of real-time monitoring of dynamic loads on the human foot. Collectively, these results confirm that the output signals reliably reflect diverse movements, thereby enabling precise assessments to support effective healthcare monitoring.

**Figure 6:**
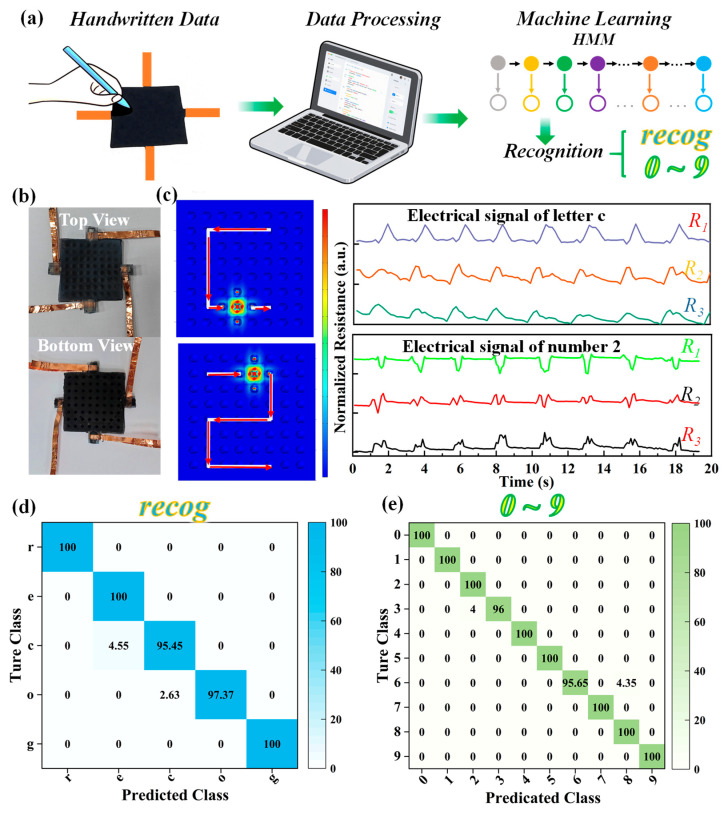
(**a**) Conceptual diagram of the designed data classification; (**b**) optical images of the sensing device (top and bottom views); (**c**) COMSOL-simulated pressure distribution maps of typical characters (letter “c” and number “2” with arrows indicating the sequential writing trajectory) and corresponding experimentally measured electrical signals; confusion matrix illustrating the results of classification for (**d**) “recog” letters and (**e**) “0–9” numbers by machine learning.

Assisted by machine learning, the self-assembled MXene/MWCNTs pressure sensors with hollow microstructured substrates could also be utilized for the automatic intelligent classification of letters or numbers. The conceptual diagram of the designed data classification is illustrated in Figure 6a, and the miniature sensor unit is arranged with an 8 × 8 pattern forming a sensing device, whose physical images of the top and bottom views are shown in Figure 6b. This sensing device possesses four copper electrodes for data acquisition via a computer, in which one electrode connects with ground, and the others are employed to form independent circuit pathways. Each letter or number is written, respectively, on the sensing device, and the pressure distribution for various characters is consistent at the contacting point because of the sequential handwriting. Figure 6c presents the simulated pressure distribution maps of typical characters (e.g., the letter “c” and number “2”) obtained using COMSOL Multiphysics Trial Version (Version 6.4), which intuitively confirm the concentrated pressure at a contacting point. More importantly, different handwritten characters generate distinct sequential electrical signals to construct the dataset including R1, R2, and R3 electrical signals, which could be effectively distinguished using a machine learning-based classification with the Hidden Markov Model (HMM) algorithm. Consequently, the corresponding sequential electrical signals of letter “c” and number “2” measured by experiments are shown in Figure 6c. A representative letter string (“recog”) is selected for further research, in which the similarity of letters, such as “e” and “c”, could lead to incorrect classification. As shown in Figure 6d, the confusion matrix for letter classification yields a final accuracy of 98.5%, which proves the effectiveness of our pressure sensors for letter classification. Similarly, the confusion matrix in Figure 6e reveals the results of “0–9” numbers classification with the accuracy exceeding 99.2%. Significantly, the high prediction accuracies for each category are achieved, which could be attributed to the strong generalization capability and impressive device performance. Thus, the self-assembled MXene/MWCNTs pressure sensors with hollow microstructures hold significant potential for practical applications, particularly in intelligent handwritten character (letters/numbers) classification coupled with the HMM machine learning algorithm.

## 4. Conclusions

In summary, a resistive pressure sensor based on self-assembled MXene/MWCNTs and novel hollow microstructure is researched systematically, wherein the MXene particles were bridged by MWCNTs to form robust, interconnected conductive networks, and hollow microstructures formed by designed molds and thermally expandable microspheres further complicate the substrates for impressive performance. Herein, with the synergistic mechanism, the pressure sensors exhibit a promising sensitivity of 2.63 kPa^−1^. In addition, a rapid response and recovery time of 340 ms and 370 ms could also be observed, respectively. A low detection limit of ~0.25% ΔR/R_0_ further extends the potential applications. These preferable performance enables good prospects for human detection, including plantar pressure, subtle swallowing actions, and joint movements. More importantly, its applications in numerical classification based on machine learning are performed, wherein an impressive accuracy of 99.2% is achieved. Collectively, our research demonstrates that the self-assembled MXene/MWCNTs pressure sensor with novel hollow microstructures provides a novel guideline for promising performance, which holds significant potential for practical applications.

## Figures and Tables

**Table 1:** Performance comparison table of relevant flexible pressure sensors.

Composition	Microstructure	Sensitivity	Range	*R* ^2^	Ref.
SWCNT/PDMS	Random	0.39 kPa^−1^	40–100 kPa	—^1^	[36]
MWCNTs/Cu/PDMS	Different size micro-domes	1.3 kPa^−1^	0–200 kPa	0.99	[37]
PEDOT:PSS/PDMS	Finger-print structure	160 kPa^−1^	0–0.5 kPa	0.978	[38]
MWCNTs/PDMS	Gaussian structure	1.77 kPa^−1^	20 Pa–30 kPa	0.99	[39]
CNT/PDMS	Sacrificial sugar templates	0.059 kPa^−1^	10−100 kPa	0.991	[40]
CNT/PDMS	Porous structure	0.247 kPa^−1^	0–175 kPa	0.997	[41]
MXene/PDMS	Sandpaper	2.6 kPa^−1^	0–30 kPa	—^1^	[42]
MXene/MWCNTs/PDMS	Semisphere nested by pyramid with TEMs	2.63 kPa^−1^	0–45 kPa	0.95	This work

^1^ “—”: Represents no corresponding data or unmeasurable value.

## Data Availability

The data that support the findings of this study are available from the corresponding author upon reasonable request.

## References

[1] Zhao W., Li K., Li Z., Wang W., Yu X., Zhang T., Yang X. (2025). Flexible Pressure Sensor Arrays with High Sensitivity and High Density Based on Spinous Microstructures for Carved Patterns Recognition. Adv. Funct. Mater..

[2] He J., Wang S., Han R., Liu Y., Gao W., Bao R., Pan C. (2025). Wide detection range flexible pressure sensors based on 3D interlocking structure TPU/ZnO NWs. Adv. Funct. Mater..

[3] Qin R., Nong J., Wang K., Liu Y., Zhou S., Hu M., Zhao H., Shan G. (2024). Recent advances in flexible pressure sensors based on MXene materials. Adv. Mater..

[4] Xu C., Chen J., Zhu Z., Liu M., Lan R., Chen X., Tang W., Zhang Y., Li H. (2024). Flexible pressure sensors in human–machine interface applications. Small.

[5] Yi F., Guo Y., Wu S., Zhu Y., Cui Z., Huang A. (2025). 3D Porous Thermoplastic Polyurethane/Carbon Nanotube@ Silver Nanoparticle Foam with Multidimensional Conductive Networks for Flexible Electronic Sensing. ACS Appl. Polym. Mater..

[6] Qin J., Yin L.J., Hao Y.N., Zhong S.L., Zhang D.L., Bi K., Zhang Y.X., Zhao Y., Dang Z. (2021). Flexible and stretchable capacitive sensors with different microstructures. Adv. Mater..

[7] Li W., Zou K., Guo J., Zhang C., Feng J., You J., Cheng G., Zhou Q., Kong M., Li G. (2024). Integrated fibrous iontronic pressure sensors with high sensitivity and reliability for human plantar pressure and gait analysis. ACS Nano.

[8] Huang C.B., Yao Y., Montes-García V., Stoeckel M.A., Von Holst M., Ciesielski A., Samori P. (2021). Highly sensitive strain sensors based on molecules–gold nanoparticles networks for high-resolution human pulse analysis. Small.

[9] Segev-Bar M., Haick H. (2013). Flexible sensors based on nanoparticles. ACS Nano.

[10] Kim H., Shaqeel A., Han S., Kang J., Yun J., Lee M., Lee S., Kim J., Noh S., Choi M. (2021). In situ formation of Ag nanoparticles for fiber strain sensors: Toward textile-based wearable applications. ACS Appl. Mater. Interfaces.

[11] Wang R., Sun L., Zhu X., Ge W., Li H., Li Z., Zhang H., Huang Y., Li Z., Zhang Y. (2023). Carbon nanotube-based strain sensors: Structures, fabrication, and applications. Adv. Mater. Technol..

[12] Li Q., Liu Y., Chen D., Miao J., Lin S., Cui D. (2021). Highly sensitive and flexible piezoresistive pressure sensors based on 3D reduced graphene oxide aerogel. IEEE Electron Device Lett..

[13] Qiao C., Wu H., Xu X., Guan Z., Ou-Yang W. (2021). Electrical conductivity enhancement and electronic applications of 2D Ti_3_C_2_Tx MXene materials. Adv. Mater. Interfaces.

[14] Yang W., Liu F., Lin Y., Wang J., Zhang C., Cheng H., Chen H. (2025). MXene-based flexible sensors for wearable applications. Soft Sci..

[15] Ma Y., Cheng Y., Wang J., Fu S., Zhou M., Yang Y., Li B., Zhang X., Nan C. (2022). Flexible and highly-sensitive pressure sensor based on controllably oxidized MXene. InfoMat.

[16] Du T., Han X., Yan X., Shang J., Li Y., Song J. (2023). MXene-based flexible sensors: Materials, preparation, and applications. Adv. Mater. Technol..

[17] Jin C., Bai Z. (2022). MXene-based textile sensors for wearable applications. ACS Sensors.

[18] Lai Q.T., Zhao X.H., Sun Q.J., Tang Z., Tang X.G., Roy V. (2023). Emerging MXene-based flexible tactile sensors for health monitoring and haptic perception. Small.

[19] Parvin N., Joo S.W., Jung J.H., Mandal T. (2025). Unlocking the future: Carbon nanotubes as pioneers in sensing technologies. Chemosensors.

[20] Lawaniya S.D., Kumar S., Yu Y., Awasthi K. (2023). Flexible, low-cost, and room temperature ammonia sensor based on polypyrrole and functionalized MWCNT nanocomposites in extreme bending conditions. ACS Appl. Polym. Mater..

[21] Nag A., Afsarimanesh N., Nuthalapati S., Altinsoy M. (2022). Novel surfactant-induced MWCNTs/PDMS-based nanocomposites for tactile sensing applications. Materials.

[22] Li X., Liang Q., Liu H., Zhao L., Sun C., Hou C. (2025). High-Sensitivity MXene/MWCNTs/PDMS Flexible Capacitive Sensor for Wearable Health Monitoring. Adv. Mater. Technol..

[23] You J., Zhang J., Zhang J., Yang Z., Zhang X. (2022). Stretchable and highly sensitive strain sensor based on a 2D MXene and 1D whisker carbon nanotube binary composite film. ACS Appl. Mater. Interfaces.

[24] Chen S., Xu J., Shi M., Yu Y., Xu Q., Duan X., Gao Y., Lu L. (2021). Polydopamine bridged MXene and NH2-MWCNTs nanohybrid for high-performance electrochemical sensing of Acetaminophen. Appl. Surf. Sci..

[25] Wang F., Yu H., Ma X., Lv X., Liu Y., Wang H., Wang Z., Chen D. (2024). A Highly Sensitive Strain Sensor with Self-Assembled MXene/Multi-Walled Carbon Nanotube Sliding Networks for Gesture Recognition. Micromachines.

[26] Xiong W., Zhang F., Qu S., Yin L., Li K., Huang Y. (2024). Marangoni-driven deterministic formation of softer, hollow microstructures for sensitivity-enhanced tactile system. Nat. Commun..

[27] Zhu M., Chen C., Yu A., Feng Y., Cui H., Zhou R., Zhuang Y., Hu X., Liu S., Zhao Q. (2025). Multilayer step-like microstructured flexible pressure sensing system integrated with patterned electrochromic display for visual detection. ACS Nano.

[28] Zhang Y., Zhou X., Zhang N., Zhu J., Bai N., Hou X., Sun T., Li G., Zhao L., Chen Y. (2024). Ultrafast piezocapacitive soft pressure sensors with over 10 kHz bandwidth via bonded microstructured interfaces. Nat. Commun..

[29] Tang R., Lu F., Liu L., Yan Y., Du Q., Zhang B., Zhou T., Fu H. (2021). Flexible pressure sensors with microstructures. Nano Sel..

[30] Li Y., Jiang D., An Y., Chen W., Huang Z., Jiang B. (2024). Wearable flexible pressure sensors: An intriguing design towards microstructural functionalization. J. Mater. Chem. A.

[31] Su S., Zhang X., Dang D., Wang Z., Tong Z. (2024). A high-performance flexible capacitive pressure sensor with 3-D printed hemispherical graded microstructures. IEEE Sensors J..

[32] Luo N., Huang Y., Liu J., Chen S.C., Wong C.P., Zhao N. (2017). Hollow-structured graphene–silicone-composite-based piezoresistive sensors: Decoupled property tuning and bending reliability. Adv. Mater..

[33] Bai Y., Zhang S., Ji J. (2024). Flexible pressure sensor decorated with MXene and multi-walled carbon nanotube composites for motion monitoring and electronic skin. J. Mater. Sci. Mater. Electron..

[34] Xia T., Yu R., Yuan J., Yi C., Ma L., Liu F., Cheng G. (2021). Ultrahigh sensitivity flexible pressure sensors based on 3d-printed hollow microstructures for electronic skins. Adv. Mater. Technol..

[35] Xiao Y., Wang K., Yu X.-D., Xu J.-J., Chen H.-Y. (2007). Separation of aminophenol isomers in polyelectrolyte multilayers modified PDMS microchip. Talanta.

[36] Abodurexiti A., Yang C., Maimaitiyiming X. (2020). High-performance flexible pressure and temperature sensors with complex leather structure. Macromol. Mater. Eng..

[37] Lee J., So H. (2023). 3D-printing-assisted flexible pressure sensor with a concentric circle pattern and high sensitivity for health monitoring. Microsyst. Nanoeng..

[38] Xu Z., Wu D., Chen Z., Wang Z., Cao C., Shao X., Zhou G., Zhang S., Wang L., Sun D. (2023). A flexible pressure sensor with highly customizable sensitivity and linearity via positive design of microhierarchical structures with a hyperelastic model. Microsyst. Nanoeng..

[39] Zhu B., Xu Z., Liu X., Wang Z., Zhang Y., Chen Q., Teh K.S., Zheng J., Du X., Wu D. (2023). High-linearity flexible pressure sensor based on the Gaussian-curve-shaped microstructure for human physiological signal monitoring. ACS Sensors.

[40] Jung Y., Lee T., Oh J., Park B.-G., Ko J.S., Kim H., Yun J.P., Cho H. (2021). Linearly sensitive pressure sensor based on a porous multistacked composite structure with controlled mechanical and electrical properties. ACS Appl. Mater. Interfaces.

[41] Zhong Y., Wu L., Gu F., Wang J., Dai S., Zhu H., Cheng G., Ding J. (2023). Negative pressure-assisted porous structure with gradient dielectrics design for linearity enhancement of flexible capacitance pressure sensor. Colloids Surf. A Physicochem. Eng. Asp..

[42] Chen B., Zhang L., Li H., Lai X., Zeng X. (2022). Skin-inspired flexible and high-performance MXene@ polydimethylsiloxane piezoresistive pressure sensor for human motion detection. J. Colloid Interface Sci..

